# “The Referee Plays to Be Insulted!”: An Exploratory Qualitative Study on the Spanish Football Referees’ Experiences of Aggression, Violence, and Coping

**DOI:** 10.3389/fpsyg.2021.656437

**Published:** 2021-04-28

**Authors:** José Devís-Devís, José Serrano-Durá, Pere Molina

**Affiliations:** Departament d’Educació Física i Esportiva, University of Valencia, Valencia, Spain

**Keywords:** umpire, sexism, abuse, racism, sport education, symbolic violence

## Abstract

Referees are essential participants in the sport of football. They are responsible for enforcing the rules and achieving the necessary impartiality for the matches. Referees are often target of hostile reactions from fans, players, and coaches. However, few studies have focused on these experiences and the strategies they use to manage them. In order to fill this gap, a qualitative interview-based study was developed to explore the experiences of a group of football referees (four males and four females) on aggression, violence, and coping. A thematic analysis was developed combining inductive and deductive processes. Results indicated that the most frequent aggressions experienced were verbal abuse. Most of the aggressions from spectators were of a sexist nature. When aggressions were considered normal by referees, symbolic violence emerged. Racist aggressions were directed to the two Moroccan participants. Problem and emotional-focused coping strategies were identified. The two most common referee responses to coaches’ and players’ verbal abuse were penalties and send-off calls. Smiling and not considering insults as a personal matter were two emotional-focused coping strategies used toward spectator aggressions. Implications for the referees’ job and well-being as well as the quality of football competitions were highlighted to reduce aggressions and violence and to help referees to cope with hostile behaviors.

## Introduction

Sport referees are essential participants in the production of organized matches and tasked with the mission of enforcing the rules and preserving fairness during the competition (Cruz, 1997; Louvet, 2011). They have a difficult and complex job with many aspects to deal (e.g., judging, making quick decisions, resolving disputes, managing the matches, and coping with abuse) to contribute to overall quality of sport competitions (Guillén and Feltz, 2011; Rix-Lièvre et al., 2014). Moreover, the referees’ role is currently being propelled by government policies worldwide since they are encouraging participation in sports (Cuskelly and Hoye, 2013).

According to the relevant role sports referees play within the sports system, recruitment, retention, decision-making, and experiences deserve a prominent space in the investigation of sports sciences and management studies (Mascarenhas and Smith, 2011; Livingston et al., 2017; Morillo et al., 2017). In fact, there is an increase interest in researching these issues in the last decade (Pina et al., 2018; Livingston et al., 2020). However, there is still a necessity of more research that especially focuses on issues of aggression, violence, and coping, particularly in football fields.

Aggression has been referred to a consequence of frustration or a reaction to a negative effect experienced when encountering aversive stimulus (Berkowitz, 1993). It is not an attitude but a behavior, which is reflected in acts committed with the intent to harm or injure. A common definition of aggression comes from Baron and Richardson (1994), which involves intent and refers to “any form of behavior directed toward the goal of harming or injuring another living being who is motivated to avoid that such treatment” (p. 7). Aggressions can be classified into *hostile* and *instrumental*, according to the primary reinforcement sought *via* the act. The first ones are addressed to produce harm upon another by its own sake, while the second type of aggressions is directed to the achievement of a subsequent goal (Baron and Richardson, 1994).

As a subset form of aggression, violence is commonly understood as a *physical component of aggression* or extreme behavior used to inflict severe harm to other people whether it ultimately causes actual harm or not (Anderson and Bushman, 2002). Thus, aggression and violence are best conceptualized as parts of a continuum with minor acts of aggression (e.g., pushing) at the low end and extreme violence (e.g., homicide) at the high end of the spectrum. However, certain types of verbal aggression aimed at children or partners are labeled as *emotional violence* due to the severity of harm to their social and emotional well-being (Allen and Anderson, 2017). Several coaching behaviors in the field of young elite sport, such as shouting, belittling, threats, and humiliation (Gervis and Dunn, 2004), when repeatedly performed are examples of emotional violence. Likewise, another form of violence without a physical component is the so-called *symbolic violence*. According to Bourdieu (1998), it is “the violence which is exercised upon a social agent with his or her complicity” (p. 167) and relies upon “the set of fundamental, prereflexive assumptions that social agents engage in by the mere fact of taking the world for granted, of accepting the world as it is, and of finding it natural because their mind is constructed according to cognitive structures that are issued out of the very structures of the world” (p. 168). Therefore, *symbolic violence* refers to subtle and indirect forms of coercion and aggression, which are considered as obvious or covert, because they are embedded in the culture and have deep consequences for persons and social groups. Authoritarian speeches in a training context, which are exclusively coach-led, shape the contours of a training process that affects subsequent relations of domination during the everyday interactions between coaches and players (Cushion and Jones, 2006). This example of symbolic violence is less direct and more imperceptible than the emotional violence, because the relations of dominance emerge with the complicity of players by taking for granted the interactions and culture embedded in such a context.

Aggression and violence are often seen as stressful events, which people cope in their everyday life. Coping is defined as “the constantly changing cognitive and behavioral efforts to manage specific external and/or internal demands that are appraised as taxing or exceeding the resources of the person” (Lazarus and Folkman, 1984, p. 141). It is a conscious, dynamic, and specific process, not a general and stable characteristic employed to control aversive situations and reduce the distress produced by them. Coping also has a transactional meaning since it is not characteristic of the situation or the individual, but a process involving interactions between a person and his or her environment.

A broad range of coping strategies used to face stressful situations has been identified due to the diversity of the situations considered. Among them, *problem-focused coping* and *emotional-focused coping* were identified by Lazarus and Folkman (1984). The former refers to cognitive efforts to redefine or change the problem located in the individual-environment relationships and to select among alternative options of action to make a change in reality. For instance, when a referee must face a coach’s verbal abuse, he or she may choose to give a technical foul, warn the individual, or walk away from him or her. These efforts to cope would be directed outwardly to alter something from the environment or inwardly to alter something from oneself. *Emotional-focused coping* refers to cognitive efforts to reduce emotional distress such as avoidance, distancing, blaming, minimizing, wishful thinking, venting emotions, and meditating. For example, if a spectator’s verbal aggression upsets a referee, he or she may feel the appropriate strategy is to minimize or ignore it.

### Aggression, Violence, and Coping Among Football Referees

Referees from different sports, such as football, hockey, rugby, and cricket, and across different continents suffered *hostile* reactions to their decisions (Fields et al., 2007; Kellett and Shilbury, 2007; Ackery et al., 2012; Webb et al., 2019). In the particular case of football referees, different types of physical and verbal assaults have been identified, mainly due to the lack of knowledge of the rules by the public and the lack of commitment by the referees (Friman et al., 2004; Simmons, 2006). Folkesson et al. (2002) observed that 63.6% of Swedish football referees had suffered verbal aggression at least one occasion either from players, coaches, spectator, and/or others. This value is close to the 60% pointed out by Cleland et al. (2017) among English referees. The most frequent aggressions experienced by Canadian referees were abusive remarks, followed by threatening and intimidation (Deal et al., 2018).

Folkesson et al. (2002) also found that younger referees were more exposed to incidents of threat and aggression than older ones, but those with more refereeing experience were exposed to more incidents of aggression than the referees with less experience. Furthermore, pessimistic referees displayed greater difficulties coping with aggressive behaviors from the spectators rather than those optimistic referees.

Violence was also observed by means of assaults (pushing, kicking, and punching), invasions of the pitch, throwing objects, and attacking their personal vehicles (Folkesson et al., 2002; González-Oya, 2006). Folkesson et al. (2002) reported that 15% of Swedish referees experienced *physical aggression* on, at least, one occasion either from players, coaches, fans, and/or others. The percentage of English referees who suffered physical abuse reaches 19% (Cleland et al., 2017). Deliberate violent conduct and physical contact with referees were, after the verbal and intimidating behaviors, the more frequent aggressive incidents to Canadian referees (Deal et al., 2018). On occasion, referees received death threats and sometimes these threats extended over a long period of time. In the case of the controversial performance of Howard Webb in the Poland-Austria match of the Euro 2008 UEFA Football Championship, the referee and his family required police protection for several months (Louvet, 2011).

Football referees used *problem-focused coping strategies* four times more than the *emotional-focused* ones (Voight, 2009), and they remained unchanged while *emotional-focused* ones decreased throughout the entire season (Louvet et al., 2009). Nevertheless, we must highlight that these strategies adapted according to the various stressors of the environmental situation, including aggressions and violence. According to Voight (2009), the most used strategy by United States referees to cope with the corresponding aggressive sources was to ‘ask fellow officials’ how to overcome the aggressive situations. Other studies from Sweden and Australia indicated that referees coped aggressions from angry players by communicating with them to restore their conduct (Friman et al., 2004; Simmons, 2006). As an example, Praschinger et al. (2011) found that half of Austrian referees from their study would expel a player because of verbal abuse (55.7%), while others would just give them a warning with yellow card (25.2%) or blue card (12.1%). Yet others chose to simply not react in such cases (7%).

The more frequently used *emotional-focused strategies* by football referees were calling for social support or denial and, to a lesser extent, avoidance tactics in order to manage spectators’ angry behavior (Friman et al., 2004; Simmons, 2006; Voight, 2009). It is suggested that referees from collectivistic Asian cultures used more *emotional-focused coping strategies* than their individualistic counterparts from Western cultures, which used more *problem-focused coping* (Kim and Duda, 2003).

Studies on aggression, violence, and coping among football referees according to gender variables are scarce. Forbes et al. (2015) were some of the few who indicated that female referees from United Kingdom endure a broader culture of abuse in comparison to their male counterparts. These authors suggested that female referees not only had to cope with the insults referees habitually receive but also had to face sexist abuse. The normalization of sexist situations by means of accepting these behaviors as just ‘part of the game’ was the *coping strategy* used by these referees. The experiences of Canadian women referee in football also showed how they confronted normalized sexual discrimination and internalized the sexism they face (Reid and Dallaire, 2019).

According to studies developed in basketball (Tingle et al., 2014), women officials experience more sexist abuse and associated coping than their male counterparts, but the worst experience they felt was the dearth of advocates and confidants that helped them to cope with this gendered abuse. The autoethnography of Schaeperkoetter (2017) on her experience as basketball referee also showed how she used the ‘ignoring’ strategy for managing sexist comments. However, the study developed by Wolfson and Neave (2007) with English football referees did not reveal differences in the *coping process* due to age, experience, or education. The authors did not search significant differences by gender because of the low number of women referees participating in their study, but they suggested the possibility of using different coping mechanisms by gender as a hypothesis for future studies. The lack of studies on ethnic and race issues related to abuse and coping among football referees reflects, according to Cleland and Cashmore (2014), the ‘color blind’ ideology, which dominates the culture of football.

As an international cultural phenomenon, football refereeing is plenty of cultural variations. Therefore, it is necessary to undertake more studies, which contribute to a deep understanding on the role and features of aggression, violence, and coping in football officiating worldwide. This paper suits the aforementioned need since its purpose consists of exploring Spanish football referees’ experiences on these issues. More specifically, the two interrelated objectives of this paper are

1. To know the types of aggression and violence referees experienced, and the strategies they used to manage these behaviors.
2. To understand the personal and contextual characteristics involved in the aggression, violence, and coping experienced by Spanish football referees.

## Methodology

A qualitative research methodology was chosen to develop this study and focused, according to Polkinghorne (2005), to the study of people’s ordinary life experiences. As a qualitative study, the purpose was interpreting and understanding different empirical materials to make the world visible (Denzin and Lincoln, 2011). In this case, understanding the ordinary game experiences on aggression, violence, and coping among Spanish football referees and the inseparable context in which these experiences were embedded. It was possible through face-to-face semi-structured interviews as sources of data considered appropriate for obtaining rich descriptions and also for interpreting experiences (Smith and Sparkes, 2016).

### Participants and Recruitment

This qualitative semi-structured interview-based study is part of a wider project on the analysis of different sport systems, especially football, from a fair play and social justice perspective. Eight football referees from the Mediterranean Coast of Spain were chosen according to a purposeful sampling procedure, which attended substantial diversity (Polkinghorne, 2005) in order to widely explore heterogeneity in the experiences under study. In particular, different gender and ethnicity, variety of football categories, and years of experience as referee were attended.

Four males and four females, ranging between 19 and 34 years of age and with 2–17 years of experience, participated in the study. The eight referees came from different football categories from baby football to professional football. All of them had experience in grassroots youth football (from 6 to 18 years of age), and in both men’s and women’s football competitions. Two referees had experience uniquely in children’s and youth games, while the rest had been promoted to higher categories of either amateur (over 18 s) or professional football. They performed in different categories at the same time. Six of them were Spanish and two were of Moroccan nationality (see Table 1).

**Table 1:** Participants’ main characteristics.

	Gender	Age	Nationality	Experience		Highest level refereed
				Years	Categories	
Trini	Female	21	Spanish	8	Grassroots and amateur football	Men’s Second Regional Amateur League
Leyre	Female	19	Spanish	7	Grassroots, amateur, and female professional football	Women’s First Division
Clarke	Male	22	Moroccan	4	Grassroots and amateur football	Men’s Second Regional Amateur League
Omar	Male	23	Moroccan	3	Grassroots and amateur football	Men’s Second Regional Amateur League
Javier	Male	34	Spanish	17	Grassroots, amateur, and male professional football	Men’s First Division (4th referee)
Lola	Female	23	Spanish	5	Grassroots, amateur, and female professional football	Women’s Second Division
Santiago	Male	20	Spanish	3	Grassroots youth football	Men’s Second Regional Youth League
Jane	Female	22	Spanish	2	Grassroots youth football	Men’s Second Regional Youth League

Grassroots football = male and female players from 6 to 18 years of age. Amateur football = male and female players over 18 years of age.

The number of participants was in between different types of qualitative studies and proposals. According to Robinson (2014), interview studies with an ideographic aim use sample sizes in a range of 3–16 participants, being narrative and life history research those with lower sampling (e.g., Carless and Sparkes, 2008; Newman et al., 2016). Regarding the present study, the final sample size was intrinsically justified to fulfill the objectives of this exploratory study on aggression, violence, and coping since eight participants were considered enough to achieve the diversity in the sampling and accomplish the thematic analysis used, as can be observed in other qualitative studies (e.g., McHugh Power et al., 2017; Devís-Devís et al., 2018).

The participants were recruited from the Federation of Football Technical Committee of Referees to which the second author of this paper was once affiliated. After gaining permission from the institution, an email was sent to all the female referees who belonged to the delegation. This decision was made due to the low number of women referees available and the difficulties expected in recruiting female participants (Deal et al., 2018). Interviews were carried out during the months of April, May, and June 2016. Most of the referees (five) were interviewed in the Committee of Referees’ office and, the same day, they received their refereeing assignments for the following weekend. An empty room offered by the Committee was used for the interviewing process. The rest of the referees (three) were interviewed in their respective workplaces in a similar space.

### Data Collection and Analysis

An interview guide based on previous literature about referee aggression, violence, and coping strategies was developed by the researchers. This guide helped them to organize the main issues surrounding the topic of study and made the questions easy to understand for their particular audience (Kvale, 2011). All the interviews were audio-recorded and lasted between 40 min and 1 h.

The interview questions were created based on the purpose of the study. Examples from the interview guide were the following: “Have you ever feared for your physical integrity?”; “Under what circumstances?”; “Which insults bothered you the most?”; “How do you deal with the insults received from the stand?”; “How do you react to the insults that come from the players?”; and “Is your reaction different depending on the type of insults and the age of the players?.” Although the interviews were structured to some degree, they were dynamic and evolved according to their development (Kvale, 2011).

A thematic analysis was developed in order to identify the patterns of meaning across the qualitative dataset and means reflecting heterogeneous experiences about similar realities (Braun et al., 2016). It comprised inductive and deductive processes. The former one proceeded bottom-up to draw inferences about the data in a process that evolved from descriptive responses to categories and themes. Then the concepts of aggression, violence, and coping were used to classify deductively the themes and connect them with the objectives of the study.

Specifically, researchers repeatedly read data in a preliminary stage, annotating in the margins. The systematic reading explored similarities and differences as well as established relationships within the transcribed interviews and the existing literature on referees’ experiences of aggression, violence, and coping. This process allowed categories and later higher-order themes to surface, which were then classified for meaning-making. It evolved in such a way that these categories were constantly being redefined by the researchers until they were organized into the three main theoretically driven sections of results and discussion epigraph.

In terms of the assessment of the research trustworthiness, the principles of Lincoln and Guba (1985) were followed, which was seen as a parallel framework to positivist notions of rigor. Credibility is the equivalent criteria to internal validity, confirmability to objectivity, dependability to reliability, and transferability to the generalization of results. In order to achieve trustworthiness in this study, several strategies were adopted. For instance, interviews were tape recorded and transcribed in the briefest time possible (credibility), the interviewer was also the transcriber (dependability), and transcripts were returned to each participant to confirm the data recorded and add new information if necessary (credibility). Moreover, the participants were selected according to substantial diversity or maximum variation criteria (transferability), codification was shared by the researchers (confirmability), quotes were used to provide detailed descriptions and evidence (credibility, confirmability, dependability, and transferability), and structural coherence was searched in the research report (credibility).

### Ethical Issues

The wider research project from which this study belongs to and the materials and procedures used in this paper were approved by the Ethics Committee of the University of Valencia. The participating referees read and signed a written informed consent that was e-mailed in an initial contact. This document included the purpose of the study and other conditions applied to guarantee their confidentiality and privacy. Despite the impossibility of achieving complete hermetic confidentiality, the identity and location of the interviewees were protected so that they could not be identified. Their names, locations, and the places mentioned during the interviews were modified, while preserving the experiences and social contexts in which they happened. To avoid any alteration of their experiences and facilitate their recognition in the transcriptions, they were given the opportunity to choose their own pseudonyms and to make other changes themselves. Approval was obtained electronically from the participants, previously to the interviews, by means of a resent e-mail that incorporated the signed informed consent as an attachment.

Special attention was given to the asymmetric informants-interviewer relationship and the potential situation of referees’ vulnerability in publicly disclosing their experiences, as suggested by specialized literature (Kvale, 2011). Talking about their experiences of violence was not a trivial matter because certain memories of physical and verbal aggressions could have immediate or subsequent effects on the persons interviewed. Thus, striving to create a balanced exchange of information, *as a fair trade* (Sparkes, 1998), the interviewer, and second author of this paper, shared with the interviewees his own personal experience of physical aggression from his time as a football referee. This sharing of experiences contributes to build an empathetic relation between interviewer and interviewees, which improve rapport and quality of data. However, interviewer is also aware and reflexive upon his role during the interview process as knowledge producer.

## Results and Discussion

### Aggressions: Sexism and Racism on Stage

The hostile aggressions intended to produce harm were the predominant type experienced by football referees. This result differs from the instrumental aggressions, which are prevalent among the athletes participating in the matches (Pedersen, 2007). The most frequent hostile aggressions experienced by referees in our study were verbal abuse coming from players, coaches, and the public in general, in a similar way as indicated in previous studies (Folkesson et al., 2002; Friman et al., 2004; González-Oya, 2006; Louvet, 2011; Warner et al., 2013; Cleland et al., 2015; Forbes et al., 2015). Foul language, such as anonymous insults and threats from the crowd on the field or in the stadium, were the common verbal aggressions suffered by referees. As Clarke pointed out, “Very often they are insults like ‘son of a bitch, clumsy and dumb,’” with a sexist charge. A real example of these aggressions came alive in the following quote: “Son of a bitch, mother fucker! Your wife is fucking and you are refereeing, stupid moron!” “This is daily life (…)” (Javier).

This sexist insult experienced by Javier, a male referee, was an example that differed from the sexist verbal aggressions of women referees because this insult was not directed at Javier but at his supposed partner. On the contrary, women referees were the direct targets of sexist behaviors from spectators, as witnessed by all the women referees from our study, especially in matches with male players. Some discrimination and prejudice came from older men who made reference to traditionally female roles when they criticized the referee’s job, as indicated by two female referees:

Spectators criticize us a lot, especially older people. One of them was criticizing me all the time saying, ‘Go home and clean!’ and other sexist comments like that (Jane).

Older people are old fashioned with their comments. They always criticize us by stating traditionally women roles (Leyre).

However, young men’s sexist behaviors were more hypersexualized and objectified when directed at women football referees. It was no different than other facets found in the wide field of sports. For instance, many times sports commentators and newspapers focused their attention on the athletes’ suits and outfits with sexual overtones (Hargreaves, 2000). Female figures, such as singers, cheerleaders, players, and policewomen, were sexualized in football stadiums (Jones, 2008), and this also happened with female referees. In fact, all four female referee participants mentioned different situations in which they were considered as objects or a reference was made to their physical appearance and/or sexual performance. This was an example provided by Trini:

‘When you finish, we’ll make you a Bukkake! (…) Hey, will you fuck him at night? Then you stay and fuck?’… I know all that. Sometimes when the captain of a team comes to talk to you and touches you on the back, people say ‘hey, has he touched your ass? Now we know how they win!’

The act of objectification illustrated in this quotation suggested an absence of empathy toward female referees by some male spectators. This probably emerged, according to Messner (2002), in relation to strongly homosocial interactions in the social construction of masculinities in the football fan subculture. That is to say, the attitudes and feelings that men share to build their masculinity within this subculture resulted in the rejection of women and the suppression of empathy toward them.

Probably due to the social context on the football fields, our female referees also suffered sexist insults from female spectators, and these were the verbal aggressions they disliked the most. The following two quotes exemplify this issue:

Once when I was an assistant, a woman spectator said… ‘Leave the flag and go be a catwalk model!’ (…) Those are the insults that bother me the most because they are sexist comments from women (Lola).

Oh woman! They tell me: ‘Go iron! Go cook! Your job is in the kitchen! Your macaroni is going to stick to the pan!’… They are telling me that football is not for women. This is typical. That is what surprises me the most… Then, we cannot complain about macho culture (Trini).

These quotations illustrate how female spectators imitated the bravado and macho language of citing traditionally feminine roles instead of the sexual reification used by men. These females tried to behave like the male spectators because they wanted to become authentic football fans, which compensate the extended idea in the fandom that it was not possible to be a woman and understand football (Woodhouse and Williams, 1999; Llopis-Goig, 2015). Therefore, as Jones (2008) suggested, it would be a strategy for renegotiating their female identities in a homosocial territory.

Referees also suffered aggressions from players and coaches during football games. These aggressions were primarily offensiveness, rudeness, or the use of obscene language or gestures. Curiously, the sexist behaviors directed toward women referees by male players and coaches usually happened after the matches in the form of friend requests through social network applications. Jane referred it in the following way: “They would ask me to join a lot, yes. Especially the boys. Especially players. When I finish a game, I might find 13 requests on Instagram… and I block everyone (laughs).” This seems a subtle technological way to sexualize women referees by young men, which adds to other traditional forms documented by Jones (2008) in football culture.

Some of the verbal aggressions had a racist character and, in this study, they were experienced by the two Moroccan referees. For instance, Clarke mentioned one match in particular:

Once they [spectators] told me: ‘Fucking Muslim! You must have jumped the fence of Melilla!’ [an immigration wall on the North-African Spanish border] (…) Sometimes I still hear ‘fucking Muslim’, but not as much as that day. It was too much.

This quote shows a particular anti-Muslim sentiment, which should be contextualized within the increase of migration of people from Nord-African countries into Spain, reflecting a form of cultural racism also observed elsewhere in football fields (Cleland and Cashmore, 2014; Llopis-Goig, 2015).

Sometimes the referees felt that the racist insults were uttered to bother them and interfere with their job without any real knowledge of their ethnicity, race, or nationality. The following quote from Omar apparently shows how insults are made with instrumental instead of hostile purpose, because are not addressed to produce harm but to interfere with referee’s job:

There are people who cry out racist insults and do not know where I come from. For instance, they tell me: ‘Hey, this is football not the cricket you play in your country!’ And cricket is played in India and not in Morocco the place I come from… Many insults make no sense.

Nevertheless, the ironic and joking attribute of this verbal aggression is an example of a “minorization” process, characterized by ascribing otherness meanings to persons who belong to social minorities (Müller et al., 2007).

### From Fans’ Verbal Aggression to Violence

During the interviews, some referees tried to justify the fans’ aggressive behaviors. For instance, Leyre said that she understood the crowd’s verbal aggressions because “everyone [fans of both teams] wants to win” while Santiago attributed these aggressions to the followers’ “overenthusiastic” attitude and deception emerged when referees’ decisions went against their team. These explanations were similar to the manner of understanding human aggression presented by Berkowitz (1993) as a consequence of frustration or a reaction against an aversive stimulus. Even, Omar, another referee, assigned a social therapeutic character to the audiences’ verbal aggressions, probably echoing popular ways of understanding dirty language in football matches, as he said:

To be honest, I don’t care [about verbal aggressions] (…) Sometimes, I think … If these people come here to vent on me, if they release their contained anger on me and when they get home are teddy bears… then that is great, no domestic violence…

However, with these views, referees trivialized and assumed the normalization of aggressions by fans as being part of the game. Javier illustrates this in the following comment: “This is daily life (…) It is like someone who goes to the sea knowing there might be jellyfish there. It is part of the game.”

These assumptions, present in the comments of the eight referees about fans’ verbal aggressions toward them, supported two of the key characteristics of symbolic violence, as Bourdieu (2000) pointed out. First, aggressions were presupposed to be part of the natural order of the sport of football. And second, this logic was assumed by the persons who were the target of these aggressions. As Bourdieu (2000, p.1) emphasized, symbolic violence was considered as “a gentle violence, imperceptible, and invisible even to its victims.” For the referees themselves, although the insults received violated their position as judges, they were not interpreted as personal attacks and the referee became an object that everyone could and should despise and insult. Therefore, symbolic violence was observed among the referees in our study when they assumed the idea that everyone might belittle and insult them during football matches because verbal aggressions were normal or natural within this context. In doing so, they not only internalized this violence into their cognitive structures but also gave rise to and legitimized this violence in the first instance, as happened in previous sport studies (Cushion and Jones, 2006; Beltrán-Carrillo et al., 2012).

Beyond symbolic violence, four referees also experienced physical aggression such as having cans, bottle stoppers, and lighters thrown at them. One participant told us that his bicycle was stolen by a coach and those players and spectators had spat on him. Several of them mentioned they had witnessed different types of quarrels and fights between players, relatives of players, and football fans from different or the same team during their matches. One of these quarrels was recounted by Santiago as follows:

I saw a fight between a mother and a father in the stands. I was at a baby football match and the kids were crying near the fence instead of quarreling with each other. Some were shouting that this was football and not fight. And until the *guardia civil* (police) was called, they did not stop.

Although the violence described in this quotation was not directly addressed to Santiago, this type of situations may be perceived with fear by the referees because they can finally be the target of the violence. This is the case of Trini who referred to the fear experienced when witnessing a fight, due to the uncertainty of whether they would hit her after the quarrel:

For instance, this weekend … a fight started in the stands… and I thought that in any moment they would turn and hit me. You never know how people will react […] and they may think the referee is the culprit.

These situations indicate that an aggressive atmosphere generated in the stands may trigger potentially violent situations on the field (Sánchez-Romero et al., 2020), which may be addressed toward referees.

All the same, the worst experiences of violence against referees shared by our participants were direct physical aggressions such as being pushed, having a ball kicked into their stomachs by players, and being punched in their face by spectators. A recent assault suffered by Omar was described as follows:

Two months ago, a group of four or five people was waiting for me at the end of the match. One of them caught me from behind and another one punched me… I called the *guardia civil* (police) and they arrested one of them. Now we are in court. I have a medical report about my injuries and (…) my ears still ring because of that incident.

These attacks could have been very dangerous and usually involved a group of several offenders. Identification of the culprits was complex, and the possibility of serious injury increased considerably. Trini also stated that, after suffering a physical assault, the police had difficulties in identifying the offenders. Similar to Cross (1998), these experiences showed how vulnerable referees were to such aggressions and how easy it was for their offenders to go unpunished for their felonies. Thus, work and mediation from governing bodies at both local and national level is absolutely necessary in order to reduce these unpleasant situations (Webb et al., 2019).

### Coping Strategies

The referees from our study chose different coping strategies to deal with aggressions and violence depending on the seriousness of the acts and who the aggressors were. In fact, each group of aggressors might provoke a wide diversity of coping strategies as seen previously with basketball referees (e.g., Anshel and Weinberg, 1999). However, in the following sections, the results of this study show how football referees used problem-focused strategies linked to coaches’ and players’ aggressions and how they used emotional-focused strategies linked to spectators’ aggressions.

#### Problem-Focused Strategies for Coaches’ and Players’ Aggressions

The referees in our study used a variety of coping strategies when aggressions came from players and coaches because they were responsible for the development of the game and the strategies addressed the problems that had to be changed. Indeed, problem-focused strategies have been observed as the most commonly used strategies by football referees (Voight, 2009). The two most common referee responses to verbal aggressions made by coaches and players were penalties and send-offs that, according to Praschinger et al. (2011), were applied depending on the offensive content of the words used by players and coaches.

In particular, the majority of referees in our study (six out of eight) indicated that they sent off players or coaches if they were insulted, that is to say, if offensive, rude, or obscene language or gestures were used, as indicated in rule 12 of the FIFA regulations. Clarke explained it as follows: “I show a red card with just the slightest ‘you dumb’ or ‘you silly’ or ‘naughty’… With the minimum insult I hear…” Likewise, Lola said: “An insult is a red card and that’s all… An insult is an insult, even with gestures. Or threats… And from coaches the same, expulsion.”

This stringent way of rule application to face aggressions responded to a *rulebook refereeing style* because, according to Grunska (2011), referees employed the rules in a corrective manner without realizing that most of the rules were the subject of interpretation in each situation. Conversely, the responses to insults from the remaining two referees depended on many factors, such as the harshness of the insult, the moment of the match, the behavior of the player during the match, and the mood in which they were in at that moment. They expressed their thoughts as follows:

I book or send off a player depending on the insult. If it is rude enough then I show a bad face and send the player off. If it is not severe, then I tell him or her ‘take it easy guy’. It depends on the situation, the moment and other issues (Omar).

What is an insult and what is rudeness? Some expressions are clear [such as]… ‘Son of a bitch’ but… ‘Fuck off’… Is it an insult or rude behavior? It depends on the match, your mood […] If it happens in the first minute, then you book him or her (Javier).

As observed in these quotations, the two referees were more flexible in the application of the rules by following a *teacher style* characterized, according to MacMahon et al. (2015), by explaining the decision to the players to educate and prevent them from breaking the rules again. It is worth noting that, when the insult came from a child who participated in baby football or a young football category, the strategy used by most of the referees was to recommend to the coach that the child should be replaced and thus avoid sending him or her off. However, Trini was the only one who differed from her colleagues and was stricter when it came to children:

I am very strict about it. If it is a 10 years old child who insults me, I also send him or her off, but with good manners…. If they say, ‘Are you blind?’… If they insult me, I show a red card.

These results indicate that, beyond the personal philosophies behind the refereeing styles, the ways in which referees coped with aggressions corresponded to two contrasting positions, differing by the game conditions operating each time. The first is labeled as *rule administration* and is context-free of game factors other than the rule application, while the second one is known as *game management* and is context-dependent on different game factors (e.g., youth categories, the type of insult, and the moment of the match) for the good of the game and the fairness of the match (Unkelbach and Memmert, 2008).

Nevertheless, although most of our referees used send-offs depending on the context and situation, this option has proven to be problematic in several studies. According to Folkesson et al. (2002) and Friman et al. (2004), this tactic might generate feelings of injustice and uncertainty in players, coaches, and the public. These feelings might even increase the possibility or risk of aggression, especially when referees’ decisions were completely opposite to the views of the players, coaches, and spectators (van der Meij et al., 2015).

Referees managed the abuse that stem from the players and coaches by using the FIFA regulations. However, when they came from the public, aggressions were dealt with by asking the police to safeguard their personal integrity or report the aggression, as also documented in other studies (Friman et al., 2004; González-Oya, 2006).

#### Emotional-Focused Strategies for Fans’ Aggressions

Referees used very different coping strategies when hostile aggressions came from the crowd in comparison to the strategies used with coaches and players. They handled the aggressions by not responding directly as they might do when the aggressions arise from the participants in the match. Trini, a female referee, explained it this way:

You cannot address them [crowd people]. You cannot punish them, you cannot get them off of the field, you cannot do anything at all. They can do what they want and [as a referee] you have not got the resources to defend yourself from them and their hostility.

This characteristic of the fans’ aggressions was typically linked to the emotional-focused coping since nothing useful could be done to change the situation. As Lazarus (1993, p. 238) pointed out, emotional-focused efforts offer the best coping option under these circumstances. In fact, he said that the use of “rational problem-solving efforts can be counterproductive” and “even likely to result in chronic distress when they fail.”

In the face of verbal abuse from the public, referees in our study employed two main coping strategies: (a) ignoring and considering the aggressions to be non-personal attacks and (b) smiling at the spectators. The first one was the most common strategy mentioned by the participants as well as other studies (Friman et al., 2004), probably because they preferred to focus on what was happening in the match. Lola interpreted it as follows: “I behave as if I were deaf, ignoring the crowd. If you pay attention to the words that come from the public, you risk losing your concentration in the match.”

In fact, several studies show that verbal assaults produce a loss of concentration, performance, and motivation in football referees (Folkesson et al., 2002; Friman et al., 2004). However, in other occasions our referees simply ignored these aggressions because they assumed the aggressions came from people with a lack of sports education, as indicated by Trini: “Am I really taking seriously an insult from someone who is a football illiterate?… It is not personal… They always say the same thing to the referees.”

This quotation clearly illustrates the strategy of ignoring their aggressors’ comments to avoid losing their concentration on the job. It also shows how referees did not consider the insults to be personal in nature, a strategy also identified by Friman et al. (2004). In brief, verbal aggressions, especially those that came from the spectators, were interpreted by our participant referees as normal and trivial behavior at football matches to justify or otherwise excuse these spectators’ aggressions. The referees attributed the crowd’s verbal aggressions to what referees symbolize and not to something serious or personal. However, the acceptance and normalization of this situation included the sexist and racist aggressions detailed in ‘Aggressions: sexism and racism on stage’ section and supported patriarchy and race discrimination, which dominates football fields (Cleland and Cashmore, 2014). It also reinforces the idea that football is an eminently male space and a game for white Caucasians (Forbes et al., 2015; Llopis-Goig, 2015).

The second strategy adopted by referees was to smile during public disagreements and insults. This strategy was not prevalent in other studies and might be construed as a covert way to interact with the audience and demonstrate the referees’ dissatisfaction and shame to the spectators in an indirect way. The football crowd was characterized by lacking self-critique and football knowledge when focusing on the actions of the team they support as fans. Clarke expressed this in the following terms: “Sometimes spectators tell you ‘Hey referee, that’s not a foul’, when it was a hand ball. Then I smile and that’s all. You cannot address the public and explain your decision.”

The public might undermine the referee with offensive words, but the referee should not address the crowd during the match. In this situation, smiling became the interactive way referees showed the audience they did not agree with their words, as explained by Trini:

When people insult me, I smile, and it fucks with them a lot. They say, ‘look at her, she is smiling!’ They think I should be furious because of their words and answer back. But I turn back and smile and then they get pissed off.

According to Freedman et al. (1997), tolerating strong words with humor might help people, in this case referees, to overcome situations otherwise impossible to accept. Indeed, humor is a resilience mechanism adopted for different populations in order to cope with stressful or traumatic situations and improve their psychological well-being (Kuiper, 2012). However, in this case, smiling went beyond the self-defense emotional-focused strategy used by referees to play the role of a non-verbal message to tell the crowd their insults were harmless to them.

To be sure, this qualitative interview-based study on aggression, violence, and coping is not without limitations. The four female and four male referees who participated in the study do not represent proportional numbers of the Regional Committee of Referees. According to Smith and Sparkes (2016), the emphasis is not on large and representative numbers but in getting close to people, looking in detail their experiences, and responding to the study objectives. Moreover, the strength of the results is more theoretical and intensive than representative and extensive of the whole football referees’ population of the Regional Committee. The experiences of these referees are of great importance due to a lack of studies specifically focused on their participation in Spanish organized football and elsewhere. The role of gender and nationality/ethnicity is not analyzed in depth due to the explorative nature of this research and requires further attention in the future because these professionals may face unique experiences, as indicated in other studies (e.g. Cuskelly and Hoye, 2013; Tingle et al., 2014; Pina et al., 2018). Further quantitative studies may enhance the relevance of these results by an extensive perspective supported by a representative sample of Spanish football referees.

## Implications

The results of this paper portray a scenario in which referees are vulnerable within the social institution of football in Spain. As such, different implications for the referee’s job and well-being are highlighted.

First, the reinforcement of referees’ education with psychological preparation is a key issue for improving the well-being and the professional role of referees. It consists of providing psychological resources useful for day-to-day refereeing (Guillén and Feltz, 2011) as the basis for changing referees’ appraisals of aggression and violence, thus reducing their intensity (Anshel and Weinberg, 1999). This was achieved through communication skills, mental training, or cognitive skills for understanding and reading the complex social phenomena of football. It is presumed that these issues will help referees to gain respect and self-confidence, imperative to prevent, reduce aggression and violence, and consequently empower them with effective coping strategies. For instance, communication skills are vital to reduce stress and aggression that stems from interactions with players, coaches, and other referees. Moreover, coping strategies from successful referees or based on empirical evidence should be passed on, through explicit teaching, to inexperienced referees for gaining new resources and psychological support (Simmons, 2006). Learning and improving mental skills (muscle relaxation, imagery, biofeedback training, etc.) may contribute to achieving an optimal state before games, coping with aggression and violence and maintaining adequate mental health in general (Voight, 2009; Blumenstein and Orbach, 2014). Moreover, a keen knowledge of football as a sport and social phenomenon may allow referees to observe and interpret players’, coaches’, and fans’ behaviors, so as to better cope with aggressions and violence during the matches and their professional career (Simmons, 2006; Voight, 2009).

Second, the improvement of the quality of competitions, especially addressed to reduce aggressions and violence, which are intertwined with sexism and racism, is a complex and multifaceted phenomenon. Thus, a multi-institutional, continuous, and varied strategy to prevent these behaviors and to increase respect for referees could yield positive results. As part of it, wide social campaigns have been a focus of interest. These campaigns have granted some positive changes regarding the attitudes of stakeholders, but they have been proven unsuccessful in gaining a change in mass media behavior (Brackenridge et al., 2011; Cleland et al., 2017). Although broad social campaigns are still necessary, local, regional, and national sports institutions also have the responsibility of promoting respect and preventing aggressions against sports referees. For instance, sport’s organizations, such as Federations and clubs, have traditionally increased security and applied disciplinary actions to deter aggressions and violence, but these approaches in isolation have proven to be insufficient in promoting further respect for referees (The FA, n.d.; Roomey, 2017). Therefore, being pedagogical on aggression and violence during the broadcasting of football matches may play an important role in society as a whole, and it deserves a special role for fans education if the respect for referees is emphasized. Mass media reaches vast audiences and has the potential to make a huge social impact (Puertas-Molero et al., 2019).

Third, the education of the different agents involved in the sports competition is another aspect of marked interest, especially parents, referees, coaches, players, managers, and organizers who exert a prevailing influence in the socializing process of football competitions and trainings (Vella et al., 2013; Cassidy et al., 2016). The education of coaches and referees will favor the promotion of respect and their communication skills with other agents of football culture (González-Oya, 2006). Parents also possess a great potential here since they can influence in the socialization of young players, depending on their own behavior (positive or negative) during trainings and competitions (Elliott and Drummond, 2015). Finally, managers and organizers should play a facilitator role in elaborating policies for improving the quality of competitions and collaborating with parents, players, coaches, and mass media for reducing aggression and violence in football.

Fourth, physical education at schools deserves a special role in the multi-institutional approach to improve quality of competitions and promote respect in any sport, but particularly in football. Young students are the future generations of football players, coaches, referees, organizers and fans. In this regard, there is one particular pedagogical model used in physical education with special relevance for the respect of umpires in sports and football referees. This pedagogical model is called as ‘Sport Education’ and was created by Siedentop (1994) to enhance students’ experiences of authenticity in sport beyond the predominant sport-technique based approach that often reduces sport to something trivial. As research demonstrates (Siedentop et al., 2011; Farias et al., 2016), this model appears particularly helpful by introducing the referees’ position and promoting respect for referees’ decisions as part of the model while also including a referees’ fair play contract, student participation as judges, knowledge of rules, and decision-making as part of a good game.

In summary, referees’ job and well-being, as well as quality of football competitions should be the center of attention in determining the actions and policies to be carried out by different football organizations, professional psychologists and managers, and other institutions, which have the responsibility to educate citizens.

## Data Availability Statement

The raw data supporting the conclusions of this article will be made available by the authors, without undue reservation.

## Ethics Statement

The studies involving human participants were reviewed and approved by the Ethics Committee of the University of Valencia (Spain). The patients/participants provided their written informed consent to participate in this study.

## Author Contributions

JD-D and JS-D: conceptualization, methodology, and analysis. JS-D: data gathering. JD-D and PM: supervision. JD-D, JS-D, and PM: writing and review. All authors have read and agreed to the published version of the manuscript.

### Conflict of Interest

The authors declare that the research was conducted in the absence of any commercial or financial relationships that could be construed as a potential conflict of interest.
